# Adenosine A_1_ Receptor-Mediated Attenuation of Reciprocal Dendro-Dendritic Inhibition in the Mouse Olfactory Bulb

**DOI:** 10.3389/fncel.2017.00435

**Published:** 2018-01-15

**Authors:** Kristina Schulz, Natalie Rotermund, Katarzyna Grzelka, Jan Benz, Christian Lohr, Daniela Hirnet

**Affiliations:** ^1^Division of Neurophysiology, Institute of Zoology, University of Hamburg, Hamburg, Germany; ^2^Department of Physiology and Pathophysiology, Medical University of Warsaw, Warsaw, Poland

**Keywords:** olfactory bulb, neuromodulation, adenosine receptors, A_1_ receptor, reciprocal inhibition, reciprocal synapse, dendro-dendritic synapses

## Abstract

It is well described that A_1_ adenosine receptors inhibit synaptic transmission at excitatory synapses in the brain, but the effect of adenosine on reciprocal synapses has not been studied so far. In the olfactory bulb, the majority of synapses are reciprocal dendro-dendritic synapses mediating recurrent inhibition. We studied the effect of A_1_ receptor activation on recurrent dendro-dendritic inhibition in mitral cells using whole-cell patch-clamp recordings. Adenosine reduced dendro-dendritic inhibition in wild-type, but not in A_1_ receptor knock-out mice. Both NMDA receptor-mediated and AMPA receptor-mediated dendro-dendritic inhibition were attenuated by adenosine, indicating that reciprocal synapses between mitral cells and granule cells as well as parvalbumin interneurons were targeted by A_1_ receptors. Adenosine reduced glutamatergic self-excitation and inhibited N-type and P/Q-type calcium currents, but not L-type calcium currents in mitral cells. Attenuated glutamate release, due to A_1_ receptor-mediated calcium channel inhibition, resulted in impaired dendro-dendritic inhibition. In behavioral tests we tested the ability of wild-type and A_1_ receptor knock-out mice to find a hidden piece of food. Knock-out mice were significantly faster in locating the food. Our results indicate that A_1_ adenosine receptors attenuates dendro-dendritic reciprocal inhibition and suggest that they affect odor information processing.

## Introduction

ATP and its metabolites, ADP and adenosine, are neurotransmitters and neuromodulators acting in virtually all brain areas (Illes et al., 1996; Burnstock et al., 2011; Burnstock, 2013). Whereas ATP serves as fast neurotransmitter, ADP and adenosine rather modulate neuronal activity, e.g., by adjusting the excitability of neurons or affecting synaptic transmission. Often, ATP is released as cotransmitter during calcium-dependent exocytosis (Pankratov et al., 2006; Zhang et al., 2007; Thyssen et al., 2010), activates postsynaptic receptors and is then degraded by extracellular enzymes to give rise to ADP, AMP and adenosine (Dunwiddie et al., 1997; Sebastiao et al., 1999; Zimmermann, 2000; Zimmermann et al., 2012). Those purine-degrading extracellular enzymes are divided into different groups, amongst others containing ecto-5′-nuclotidase (CD73) and alkaline phosphatases which catalyze degradation of purine phosphates to adenosine. In rodents, the ecto-5′-nuclotidase exhibits its highest activity in the olfactory bulb as compared to other brain regions. In addition, the activity of tissue non-specific alkaline phosphatase is very high in this brain region (Langer et al., 2008). This suggests a prominent role of purinergic signaling in the olfactory bulb, especially for ATP metabolites such as adenosine. Adenosine activates P1 receptors, which are subdivided into A_1_, A_2A_, A_2B_ and A_3_ (Fredholm et al., 2001; Abbracchio et al., 2009). Adenosine receptors are G-protein-coupled receptors with a broad range of action in the brain. The impact of adenosine on synaptic function has been apparent for decades and a growing number of studies reveals the mechanisms by which adenosine modulates neuronal performance (reviewed by Cunha, 2008; Ribeiro and Sebastiao, 2010; Dias et al., 2013; Köles et al., 2016). Stimulation of A_1_ receptors, e.g., has been shown to suppress neurotransmitter release by inhibiting presynaptic calcium entry via voltage-gated calcium channels (Mogul et al., 1993; Yawo and Chuhma, 1993). Adenosine also elicits postsynaptic effects, where A_1_ receptor stimulation results in an activation of K^+^ channels and thus leads to hyperpolarization of the postsynaptic membrane (Segal, 1982; Trussell and Jackson, 1985; Gerber et al., 1989). In contrast, activation of A_2A_ receptors facilitates transmitter release (Okada et al., 1992; Mogul et al., 1993; Wirkner et al., 2004).

**FIGURE 1:**
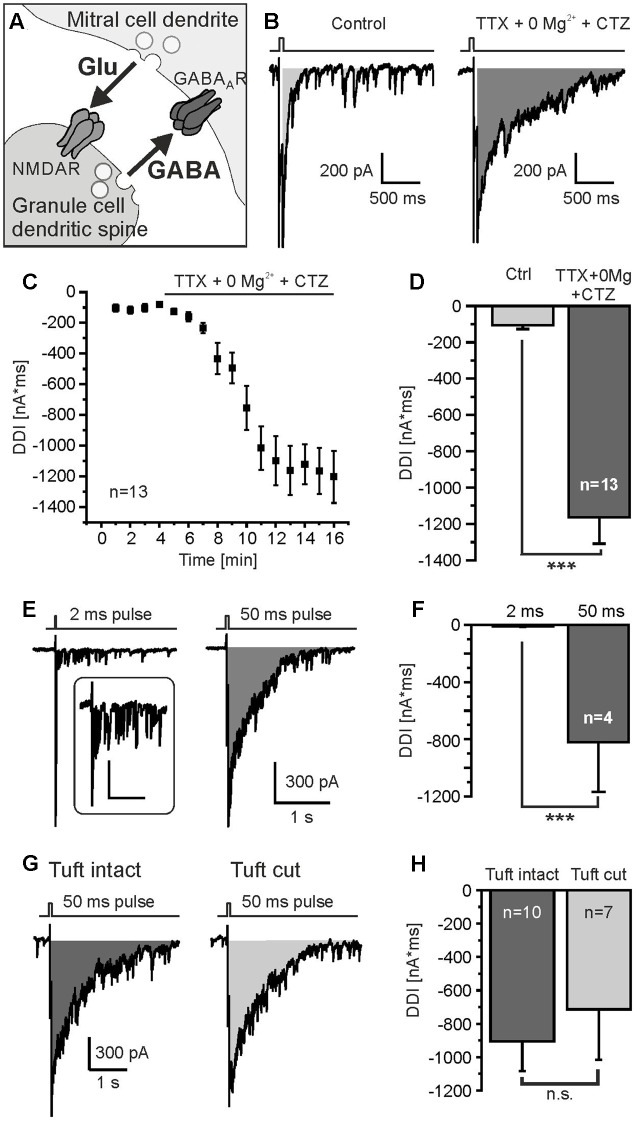
Protocol to measure DDI. **(A)** Granule cell-mediated DDI is triggered by glutamate release from mitral cell lateral dendrites and subsequent GABA release from granule cell spines. **(B)** Mitral cells were depolarized from -70 mV to 0 mV for 50 ms, as indicated by the trace on top of the current recording. Tetrodotoxin (TTX, 1 μM) was added to suppress sodium currents, Mg^2+^ was omitted (0 Mg^2+^) to increase NMDA receptor-dependent activation of granule cells, and cyclothiazide (CTZ, 200 μM) was added to increase AMPA receptor-dependent activation of interneurons. Under these conditions, DDI was strongly enhanced (right trace). DDI was assessed by calculation of the integral of the current trace within a time window of 2 s after the depolarizing pulse (gray area). **(C)** Time course of increase in DDI upon wash in of TTX, 0 Mg^2+^ and CTZ. **(D)** TTX, 0 Mg^2+^ and CTZ significantly increased DDI. **(E)** DDI evoked by a 2-ms depolarization (left trace) and 50-ms depolarization (right trace). Inset: Response to 2-ms depolarization at higher magnification; scale bars: 300 ms, 100 pA. **(F)** The current integral evoked by a 50-ms depolarization was significantly larger compared to a 2-ms depolarization. **(G)** DDI in a mitral cell with intact apical dendrite (left trace) and a mitral cell in which the apical tuft was cut during the slicing procedure (right trace). **(H)** DDI in intact mitral cells was not significantly different compared to mitral cells with cut apical tuft. ^∗∗∗^*p* < 0.001; n.s., not significant.

Most of the research on adenosinergic modulation has been done on typical axo-dendritic synapses such as synapses between Schaffer collaterals and CA1 pyramidal neurons in the hippocampus (Rombo et al., 2015) or between parallel fibers and Purkinje cells in the cerebellum (Dittman and Regehr, 1996; Zhang and Linden, 2009). Besides these unidirectional synapses, reciprocal dendro-dendritic synapses are located in some brain areas, with both pre- and postsynaptic sites located on the same dendrites (Dowling, 1968; Cheramy et al., 1981; Ludwig and Pittman, 2003; Urban and Castro, 2010). Remarkably, in the olfactory bulb, the first relay station of the olfactory pathway, dendro-dendritic synapses outnumber conventional axo-dendritic synapses by far, rendering this brain region ideal to study reciprocal synaptic transmission (Crespo et al., 2013). Within the olfactory bulb, all principal neurons, namely mitral and tufted (M/T) cells, and most interneurons are engaged in dendro-dendritic contacts (Crespo et al., 2013). The dendritic branches of the M/T cells, the single apical dendrite and several lateral dendrites, form two distinct processing units (Linster and Cleland, 2009). The apical dendrite terminates in a tuft of branches in so-called glomeruli, where it receives sensory information via axo-dendritic synapses from olfactory receptor neurons (Chen and Shepherd, 2005; Mombaerts, 2006). Besides these axo-dendritic contacts, the apical dendritic tuft forms dendro-dendritic connections with juxtaglomerular interneurons that are located in the glomerular layer and also project their dendrites into glomeruli. The lateral dendrites of M/T cells extend in the external plexiform layer (EPL) and establish dendro-dendritic synapses with axonless granule cells (**Figure 1A**) (Hinds and Hinds, 1976a,b; Mori et al., 1983; Sassoe-Pognetto, 2011; Ke et al., 2013; Bartel et al., 2015). Only recently, another group of neurons in the EPL, the parvalbumin-expressing interneurons, has been described to connect lateral dendrites of mitral cells by forming dendro-dendritic synapses (Kato et al., 2013; Miyamichi et al., 2013). Both dendro-dendritic synapses at apical and lateral dendrites are reciprocal synapses providing the morphological basis of recurrent inhibition in the bulbar network, although dendro-dendritic synapses at lateral dendrites are supposed to be much more frequent compared to their counterparts at apical dendrites (Schoppa and Urban, 2003). In mitral cell reciprocal synapses, excitation of interneurons by glutamate released from mitral cells stimulates release of GABA, which subsequently inhibits mitral cells (Nowycky et al., 1981; Jahr and Nicoll, 1982; Isaacson and Strowbridge, 1998; Schoppa et al., 1998; reviewed in Egger and Urban, 2006). The physiological role of this recurrent inhibition remains to be investigated, but is likely to play a role in odor discrimination, contrast enhancement, control of mitral cell output, synchronizing mitral cell activity, respiratory phase decorrelation and refining odor code tuning (Yokoi et al., 1995; Abraham et al., 2010; Kato et al., 2013; Miyamichi et al., 2013; Fukunaga et al., 2014; Gschwend et al., 2015; Bhalla, 2017). In the olfactory bulb, recurrent inhibition has most intensely been studied between mitral cells and granule cells (Rall et al., 1966; Jackowski et al., 1978; Isaacson and Strowbridge, 1998; Shepherd et al., 2007). Interestingly, at this reciprocal synapse, GABA release from granule cells is independent of action potential firing (Isaacson and Strowbridge, 1998; Schoppa et al., 1998; Isaacson, 1999), but is highly dependent on calcium influx into granule cell spines through NMDA receptors (Isaacson and Strowbridge, 1998; Chen et al., 2000; Halabisky et al., 2000; Isaacson, 2001; Egger et al., 2003). In contrast, recurrent inhibition between mitral cells and parvalbumin-expressing interneurons depends on calcium-permeable AMPA receptors (Kato et al., 2013; Miyamichi et al., 2013). Neuromodulators such as acetylcholine, noradrenaline and serotonin that act on mitral and/or granule cells as well as other interneurons in the olfactory bulb adjust contrast enhancement, decorrelation and signal-to-noise ratio of incoming odor information and affect short-term memory (Linster and Cleland, 2016).

Little is known so far about the impact of adenosine on synaptic transmission at reciprocal synapses. To address this issue we studied the effect of adenosine on recurrent dendro-dendritic inhibition (DDI) of mitral cells in the mouse olfactory bulb. So far, purinergic signaling in the olfactory bulb has mainly been studied in glial cells (Rieger et al., 2007; Doengi et al., 2008; Thyssen et al., 2010, 2013; reviewed in Lohr et al., 2014), whereas only few studies on purinergic signaling in olfactory bulb neurons exist (Fischer et al., 2012; Roux et al., 2015). In the olfactory bulb, release of ATP from both astrocytes and neurons as well as degradation of ATP to adenosine has been demonstrated (Doengi et al., 2008; Thyssen et al., 2010; Roux et al., 2015). The two high-affinity adenosine receptors, A_1_ and A_2A_, are highly expressed in the olfactory bulb; while A_2A_ expression in olfactory bulb neurons has been shown to be widely distributed, no study of A_1_ receptor expression on the cellular level has been published in the olfactory bulb (Mahan et al., 1991; Kaelin-Lang et al., 1999). We measured recurrent inhibition using patch-clamp recordings and studied A_1_ receptor-deficient mice in odor-related behavioral tests. Our results show that adenosine inhibits P/Q-and N-type calcium currents in mitral cells by activation of A_1_ receptors, resulting in a decrease in glutamate release. This in turn causes a reduction of excitation of granule cells as well as parvalbumin interneurons and, hence, attenuated recurrent inhibition. A_1_ receptor-deficient mice performed significantly better in a hidden food test, suggesting that A_1_ receptors might be involved in odor-related behavior.

## Materials and Methods

### Animals and Preparation

Naval Medical Research Institute (NMRI) outbred mice (without genetic modification) and A_1_ receptor knock-out mice as well as wild-type littermates (Johansson et al., 2001) were bred in the institute’s animal facility. This study was carried out in accordance with the recommendations of the European Union’s and local animal welfare guidelines. The protocol was approved by the Behörde für Gesundheit und Verbraucherschutz (Hamburg, Germany; reference number G21305/591-00.33). Horizontal brain slices were prepared from juvenile mice of both sexes (age: postnatal days 7–14) as described before (Fischer et al., 2012). Slicing artificial cerebrospinal fluid (ACSF) contained (in mM): NaCl, 83; NaHCO_3_, 26.2; NaH_2_PO_4_, 1; KCl, 2.5; saccharose, 70; D-glucose, 20; CaCl_2_, 0.5; MgSO_4_, 2.5. Mice were anesthetized with isoflurane and decapitated. Olfactory bulbs were quickly transferred into a chilled slicing artificial cerebrospinal fluid (see above). 170–200 μm thick horizontal slices of the bulbs were cut using a vibratome (Leica VT1200S, Bensheim, Germany). Brain slices were stored in recording ACSF for 30 min at 30°C and at least 15 min at room temperature before starting experiments. Recording ACSF contained (in mM): NaCl, 120; NaHCO_3_, 26; NaH_2_PO_4_, 1; KCl, 2,5; D-glucose, 2,8; CaCl_2_, 2; MgCl_2_, 1 and was adjusted to 300 mOsmol adding mannitol. ACSF was continuously gassed with carbogen (95% O_2_/5% CO_2_; buffered to pH 7.4 with CO_2_/bicarbonate).

### Drugs

Adenosine was purchased from Sigma–Aldrich (Munich, Germany); 2,3-dioxo-6-nitro-1,2,3,4-tetrahydrobenzo-[f]-quinoxaline-7-sulfonamide (NBQX), D-(-)-2-amino-5-phosphonopentanic acid (D-APV), gabazine hydrobromide (GBZ), tetrodotoxin citrate (TTX), nifedipine (Nif) and cyclothiazide (CTZ) from Abcam (Cambridge, United Kingdom); Conotoxin GVIA (CTX) and Naspm trihydrochloride from Alomone labs (Jerusalem, Israel).

### Electrophysiological Recordings

Mitral cells of the main olfactory bulb were investigated using the patch-clamp technique (Multi Clamp 700 B amplifier with PClamp 10 software, Molecular Devices, Biberach, Germany, and EPC9 with Patchmaster software, HEKA, Lambrecht, Germany). Experiments were performed at room temperature (22–24°C). Throughout the experiments, brain slices were superfused with artificial cerebrospinal fluid (recording ACSF). Drugs were applied using the perfusion system. The whole-cell configuration was employed using patch pipettes with a resistance of ∼3 MOhm. The pipette solution for the recording of DDI, self excitation and synaptic events in mitral cells contained (in mM): 4-AP, 5; CsCl, 120; EGTA, 1; HEPES, 10; TEA-Cl, 20; Na-glutamate, 10; MgCl_2_, 2; CaCl_2_, 0.5; Na-ATP, 2; Na-GTP, 0.5; Alexa Fluor 594, 0.008. The intracellular solution for the recording of spontaneous synaptic events in granule cells contained (in mM): K-gluconate, 105; NaCl, 10; K_3_-citrate, 20; HEPES, 10; EGTA, 0.25; MgCl2, 0.5; Mg-ATP, 3; Na-GTP, 0.5. Recordings were digitized at 10 kHz and bessel filtered at 2 kHz. Mitral cells were voltage-clamped at -70 mV. The serial resistance ranged from 9 to 18 MOhm, the membrane resistance of mitral cells in the presence of TTX (1 μM) ranged from 90 to 200 MOhm. Recurrent inhibition was elicited by depolarizing the cell membrane to 0 mV for 2 and 50 ms, respectively (Isaacson and Strowbridge, 1998). Due to the pipette solution containing a high chloride concentration, GABAergic recurrent inhibition was measured as inward currents. We used the integral of the inward current over a time window of 2 s after the depolarizing voltage step to quantify recurrent inhibition. To record self-excitation, the voltage step to 0 mV was shortened to 15 ms and recurrent inhibition was suppressed by 10 μM gabazine. Self-excitation was measured as the integral of the inward current over a time window of 1 s.

### Behavioral Test

A hidden food test was performed to assess the ability of the animals to find a piece of food hidden in the bedding of the cage (Yang and Crawley, 2009). Animals were trained to the food (Froot Loops, Kellog’s, Hamburg, Germany) for 2 weeks. Animals were food-deprived for 24 h before the test. A clean cage (test cage) was filled with a 4-cm layer of bedding (chipped wood), a mouse was allowed to familiarize with the test cage for 5 min and then placed in an interim cage. It was placed again in the middle of the test cage after a single Froot Loop was completely burried in one corner of the test cage. The time was measured from the moment the mouse touched the bedding until it held the Froot Loop with both paws or started to eat to assess the ability of the mouse to find the food by smelling. Animals that did not find the food within 300 s were excluded from the analysis (three wild-type mice, two knock-out mice). In this single-blinded study, the experimenter was unaware of the genotype of the mouse under investigation.

### Data Analysis and Statistics

To analyze the effect of adenosine on DDI and calcium currents, only recordings with clear recovery after wash out of adenosine were included in the analysis. Patch-clamp recordings were analyzed using Mini Analysis (Synaptosoft, Fort Lee, NJ, United States), ClampFit (Molecular Devices) and OriginPro (Northampton, MA, United States). All values are means ± standard error of the mean (SEM) with *n* representing the number of analyzed cells. IV curves were offline leak-subtracted using pClamp. The indicated membrane potential values are not corrected for the liquid junction potential of -3.5 mV. Statistical analysis was performed using Student’s *T*-test for pairs of means and one-way ANOVA with Fisher’s *post hoc* test for groups of means. The non-parametrical Mann–Whitney *U*-Test was used to evaluate the behavioral test. Means were defined as statistically different at an error probability *p* < 0.05. For depiction of data in bar graphs, all data were normalized to the control which was set to 100%, and the bars reflect the percent changes of DDI as compared to the control.

## Results

### A_1_ Adenosine Receptors Reduce Dendro-Dendritic Inhibition in Mitral Cells

To induce DDI via reciprocal synapses between mitral cells and interneurons (**Figure 1A**) we performed whole-cell patch-clamp recordings of mitral cells and applied depolarizing voltage steps (50 ms) from -70 to 0 mV as described before (Isaacson and Strowbridge, 1998; Schoppa et al., 1998). The depolarization evoked a barrage of GABAergic inputs into the mitral cell as a result of DDI (**Figure 1B**). Due to the high chloride concentration in the recording pipette solution, these GABAergic inputs were inwardly directed and added up to a large inward current which lasted several 100s of milliseconds. TTX was added in the following experiments to suppress sodium currents and hence action potentials which could interfere with the recording of recurrent inhibition. In addition, we aimed to maximize recurrent inhibition by omitting magnesium ions and adding CTZ to the external solution. This relieves the voltage-dependent magnesium block of NMDA receptors and reduces desensitization of AMPA receptors, respectively, resulting in a strong increase of DDI currents (**Figure 1B**). The postsynaptic current integral, representing the net charge transfer, was used to quantify recurrent DDI in the mitral cell (**Figure 1B**, gray area). Upon addition of TTX and CTZ and withdrawal of Mg^2+^, the recorded DDI integral of -105.9 ± 21.9 nA^∗^ms (*n* = 13) increased to -1162.8 ± 145.8 nA^∗^ms (*n* = 13, *p* = 6.5^∗^10^-6^), reflecting substantial GABA release by interneurons connected to the recorded mitral cell by reciprocal synapses (**Figures 1C,D**). We also used a shorter depolarizing pulse of 2 ms (0 mV) to mimic dendro-dendritic inhibition as evoked by a single action potential. A 2-ms pulse induced a barrage of synaptic inputs and an underlying integral of -11.6 ± 3.0 nA^∗^ms (*n* = 4) that substantially increased to -822.1 ± 347.6 nA^∗^ms (*n* = 4) upon a 50-ms pulse (**Figures 1E,F**). We used a depolarization of 50 ms in all following experiments as the current integral evoked by a 2-ms pulse appeared to be too small to be analyzed in detail. Reciprocal dendro-dendritic synapses were not only reported between interneurons and lateral dendrites of mitral cells, but also between periglomerular interneurons and the apical tuft of mitral cells in glomeruli (Toida, 2008). To estimate the relative contribution of DDI deriving from the apical dendrite, we compared recordings from mitral cells with intact apical dendrite and mitral cells in which the apical tuft had been cut during the slicing process, as visualized by Alexa Fluor 594 staining of the recorded cell (Supplementary Figure 1). DDI in mitral cells with intact tuft was slightly larger as compared to mitral cells with cut tuft, the difference not being statistically significant (*p* = 0.57) (**Figures 1G,H**). On average, DDI was -904.3 ± 179.6 nA^∗^ms (*n* = 10) in intact cells and -713.8 ± 301.9 nA^∗^ms (*n* = 7) in cells with cut tuft. The results suggest no major contribution of synapses with periglomerular interneurons to DDI. We pooled data from cells with intact tuft and with cut for all following experiments.

**FIGURE 2:**
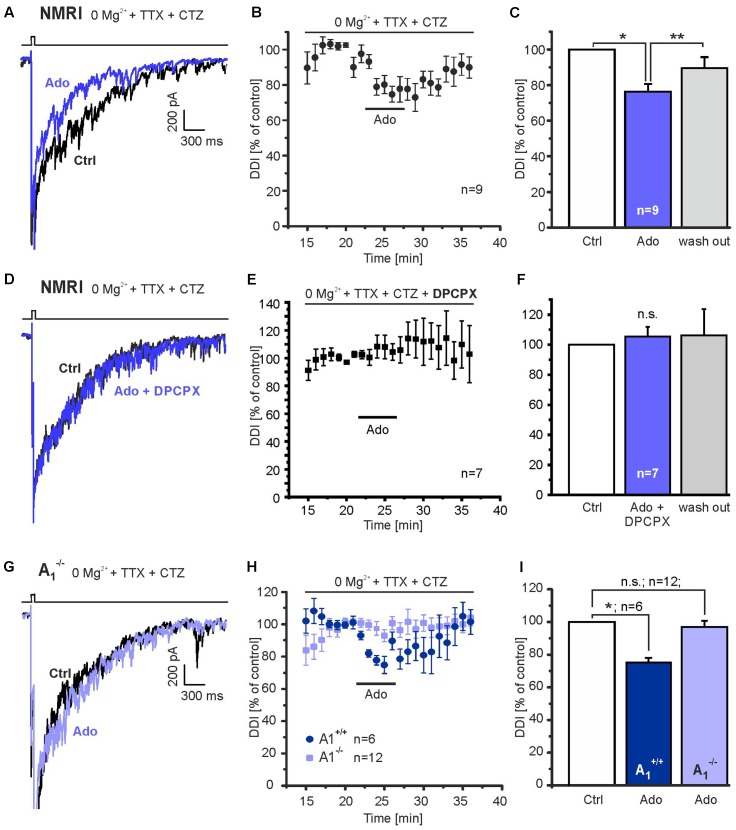
Adenosine A_1_ receptors inhibit DDI. **(A)** Application of adenosine (100 μM) reduced DDI evoked by depolarization (0 mV, 50 ms) in NMRI wild-type mice. **(B)** Time course of adenosine-evoked reduction in DDI. **(C)** The effect of adenosine on DDI was significant and reversible. **(D)** DPCPX (1 μM) entirely inhibited the effect of adenosine on DDI. **(E)** Time course of DDI in the presence of DPCPX. **(F)** Adenosine failed to inhibit DDI in the presence of DPCPX. **(G)** In A_1_ receptor knock-out mice (A_1_^-/-^), adenosine failed to reduce DDI. **(H)** Time course of DDI during application of adenosine (100 μM) in A_1_ receptor knock-out mice and wild-type littermates (A_1_^+/+^). **(I)** DDI in the presence of adenosine was significantly reduced in wild-type mice, but not in knock-out littermates. n.s., not significant; ^∗^*p* < 0.05; ^∗∗^*p* < 0.01.

We next tested the effect of adenosine on DDI. In NMRI outbred mice, which were used for most of the experiments of the study, application of 100 μM adenosine per bath perfusion reduced recurrent inhibition by 23.7 ± 4.3% (*n* = 9; *p* = 0.03) (**Figures 2A–C**). The effect of adenosine was reversible (*n* = 9; *p* = 0.0024). Reduction of synaptic activity by adenosine is mediated by A_1_ adenosine receptors in other brain areas (Mogul et al., 1993; Yawo and Chuhma, 1993). Therefore, we tested the effect of the A_1_ receptor antagonist DPCPX (1 μM). The reduction of DDI mediated by adenosine was entirely inhibited by DPCPX (**Figures 2D–F**). In the presence of adenosine, the current integral reached 105.5 ± 6.4% (*n* = 7) as compared to the control. We verified this result by testing A_1_ receptor knock-out mice for the adenosine-induced inhibition of DDI and compared the results with those from wild-type littermates. The DDI integral in A_1_ receptor knock-out mice was -1323.1 ± 164.5 nA^∗^ms (*n* = 12) and was not significantly different from wild-type littermates (-2423.8 ± 913.1 nA^∗^ms, *n* = 6; *p* = 0.286). Adenosine did not decrease the DDI integral in A_1_-deficient mice (*n* = 12; *p* = 0.543), in contrast to the adenosine-mediated reduction in DDI of 24.9 ± 2.9% (*n* = 6; *p* = 0.045) in wild-type littermates (**Figures 2G–I**). The effect of adenosine on DDI in wild-type mice was significantly larger as compared to knock-out mice (*p* = 0.0015).

### Adenosine Attenuates Glutamate Release from Mitral Cells

**FIGURE 3:**
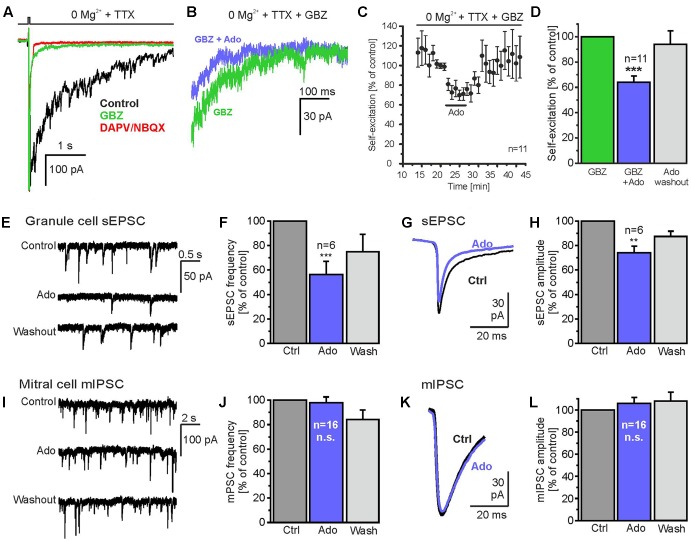
Adenosine reduces glutamate release from mitral cells. **(A)** Gabazine suppressed fast synaptic currents evoked by depolarization (0 mV, 15 ms) and unmasked a slowly inactivating inward current (green trace), which could be blocked by D-APV and NBQX (red trace) and therefore represents glutamatergic self-excitation. **(B)** Adenosine (100 μM) reduced self-excitation. **(C)** Time course of self-excitation during application of adenosine. **(D)** Adenosine significantly attenuates self-excitation. **(E)** Spontaneous excitatory postsynaptic currents (sEPSCs) in a granule cell before, during and after application of adenosine (100 μM). **(F)** Adenosine significantly reduced the frequency of sEPSCs. **(G)** sEPSCs averaged over a time of 60 s before (Ctrl) and during application of adenosine (Ado, blue trace). **(H)** Adenosine significantly reduced sEPSC amplitudes in granule cells. **(I)** Miniature inhibitory postsynaptic currents (mIPSC) recorded in a mitral cell in the presence of 1 μM TTX. IPSCs were inwardly directed due to the high chloride concentration in the pipette solution. **(J)** Adenosine had no effect on the frequency and **(K,L)** the amplitude of mIPSC. ^∗∗^*p* < 0.01; ^∗∗∗^*p* < 0.001; n.s., not significant.

Since recurrent inhibition of mitral cells by GABAergic interneurons is driven by reciprocal synapses, adenosine-mediated attenuation of transmitter release at either side of the reciprocal synapse results in a reduction of DDI. To locate the effect of adenosine at reciprocal synapses between mitral cells and interneurons we took advantage of the self-excitation of mitral cells (Nicoll and Jahr, 1982). Glutamate released by mitral cell dendrites activates local NMDA autoreceptors resulting in a slow excitatory current, which can be unmasked by application of GABA_A_ receptor blockers (Isaacson, 1999; Friedman and Strowbridge, 2000; Salin et al., 2001). We added 10 μM gabazine and 0.5 μM TTX to Mg^2+^-free ACSF to record NMDA receptor-mediated currents underlying self-excitation and evoked glutamate release from mitral cell dendrites by depolarizing the cell membrane from -70 to 0 mV for 15 ms (**Figure 3A**). Addition of gabazine suppressed fast synaptic currents reflecting reciprocal input from interneurons and unmasked an inward current with biphasic decay kinetics, consisting of a fast decaying current of large amplitude (reflecting calcium tail currents) followed by a slowly decaying inward current (**Figure 3A**, green trace). On average, the total inward current evoked by a 15-ms depolarization amounted to -746.9 ± 220.7 nA^∗^ms and was reduced by gabazine by 95.2 ± 2.5% (-23.8 ± 4.2 nA^∗^ms; *n* = 4; *p* = 0.046). Only the slowly decaying inward current was blocked by addition of the glutamate receptor blockers (**Figure 3A**, red trace) and hence represents self-excitation. The integral of the slowly decaying inward current (**Figure 3A**, area highlighted in gray) was used as a measure for the amount of released glutamate, and the effect of adenosine on this integral was tested (timeframe for analysis: 1 s). The current integral of self-excitation amounted to 19.5 ± 3.2 nA^∗^ms and application of adenosine markedly reduced the current integral by 36.0 ± 5.4% (*n* = 11; *p* = 3.7^∗^10^-4^) (**Figures 3B–D**), which suggests a prominent presynaptic inhibitory effect of adenosine on glutamate release from the mitral cell.

Granule cells receive numerous glutamatergic synaptic inputs from mitral cells. Hence, a reduction of glutamate release from mitral cells is expected to result in a decrease of the amplitude of synaptic currents in granule cells. Therefore, we recorded granule cells in whole-cell voltage clamp and measured spontaneous excitatory postsynaptic currents (sEPSC; **Figures 3E–H**). The frequency of sEPSCs was 2.8 ± 1.1 Hz (*n* = 6) and was reduced by 43.1 ± 11.7% (*n* = 6; *p* = 0.0004) upon application of adenosine, reflecting a reduction of mitral cell firing activity by adenosine (unpublished observation). To assess the effect of adenosine on the amplitude of sEPSCs, sEPSCs were averaged over a time frame of 60 s and the amplitude of the averaged current was analyzed (**Figure 3F**). 100 μM adenosine reduced the amplitude of sEPSCs by 25.9 ± 5.4% (*n* = 6; *p* = 0.0063) (**Figure 3G**), in line with the notion that adenosine reduces glutamate release from mitral cells. We also studied the impact of adenosine on GABA release from interneurons synaptically connected to mitral cells by recording miniature postsynaptic currents in the presence of TTX. Since the majority of spontaneous synaptic events in mitral cells derive from GABAergic reciprocal synapses (Supplementary Figure 2) (Egger and Urban, 2006), miniature postsynaptic currents reflect mainly miniature inhibitory postsynaptic currents (mIPSC). In mitral cells, mIPSCs occurred at a frequency of 4.16 ± 0.66 Hz (*n* = 16) (**Figures 3I,J**) and had an amplitude of -38.1 ± 3.5 pA (*n* = 16) (**Figures 3K,L**). Neither frequency (4.07 ± 0.69 Hz, *n* = 16) nor amplitude (-41.3 ± 4.8 pA, *n* = 16) of mIPSCs were altered by adenosine (**Figures 3I–L**). The results indicate that quantal GABA release from interneurons is not affected by adenosine.

### Adenosine Affects Synaptic Transmission to Granule Cells and Parvalbumin Interneurons

**FIGURE 4:**
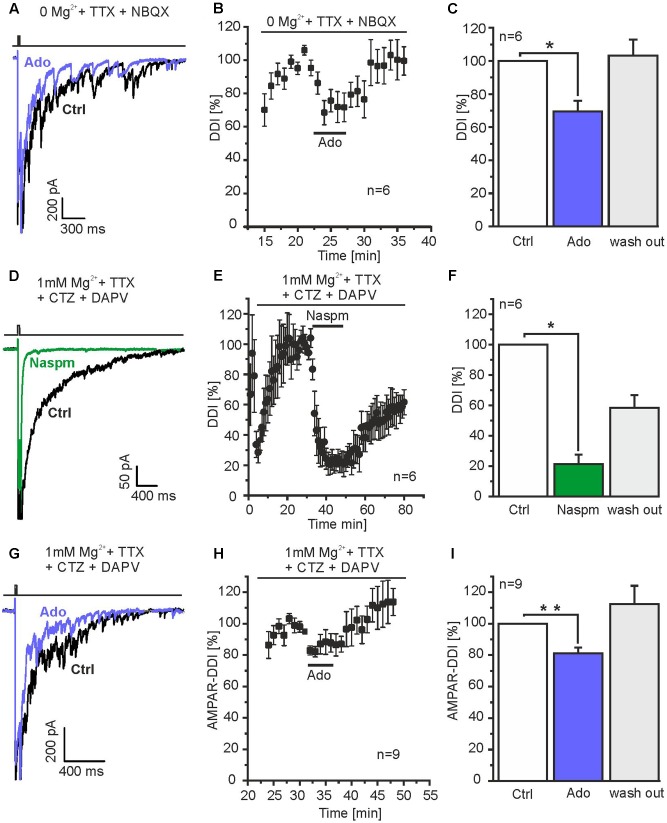
Adenosine attenuates DDI mediated by granule cells and parvalbumin interneurons. **(A)** Mg^2+^ was omitted and NBQX was added to the ACSF to isolate NMDA receptor-dependent DDI (evoked by depolarization to 0 mV for 50 ms), specific for mitral cell-to-granule cell reciprocal synapses. Under these conditions, adenosine reduced DDI. **(B)** Time course of adenosine-mediated attenuation of NMDA receptor-dependent DDI. **(C)** Adenosine significantly reduced NMDA receptor-dependent DDI. **(D)** Mg^2+^ and D-APV was added to the ACFS to suppress NMDA receptor-dependent responses, CTZ (200 μM) was added to increase AMPA receptor-dependent DDI. The specific antagonist for calcium-permeable AMPA receptors Naspm (50 μM) inhibited DDI, indicating DDI was mediated by calcium-permeable AMPA receptors as shown for parvalbumin interneurons. **(E)** Time course of Naspm-induced inhibition of DDI. **(F)** Naspm significantly inhibited DDI. **(G)** Adenosine reduced AMPA receptor-dependent DDI. **(H)** Time course of adenosine-evoked attenuation of DDI. **(I)** The effect of adenosine on DDI was significant. ^∗^*p* < 0.05; ^∗∗^*p* < 0.01.

Reciprocal dendro-dendritic inhibition in the olfactory bulb has first been described between mitral cells and granule cells, in which predominantly NMDA receptors mediate glutamatergic excitation of granule cells (Isaacson and Strowbridge, 1998; Schoppa et al., 1998). To isolate NMDAR-mediated DDI, we used Mg^2+^ free external solution containing 10 μM NBQX for the recording. Under these conditions, adenosine reduced DDI by 30.6 ± 6.4% (*n* = 6, *p* = 0.019) (**Figures 4A–C**), indicating that adenosine affects reciprocal synaptic transmission between mitral cells and granule cells.

Recently, it has been shown that reciprocal synapses between mitral cells and parvalbumin interneurons in the external plexiform layer largely contribute to DDI in mitral cells (Kato et al., 2013; Miyamichi et al., 2013). Calcium influx into parvalbumin interneurons triggering GABA release, however, is not mediated by NMDA receptors, but calcium-permeable AMPA receptors (Kato et al., 2013). In order to isolate AMPAR-mediated DDI, we blocked NMDA receptors by using external solution containing 50 μM D-APV and 1 mM Mg^2+^ and suppressed AMPAR desensitization by addition of 200 μM CTZ. Depolarization of mitral cells resulted in DDI that was inhibited by 78.8 ± 6.2% (*n* = 6; *p* = 0.019) in presence of Naspm, an antagonist of calcium-permeable AMPA receptors (Droste et al., 2017). This suggests that under these conditions, DDI was mainly mediated by calcium-permeable AMPA receptors and, hence, parvalbumin interneurons (**Figures 4D–F**). The AMPA receptor-dependent DDI was reduced by 18.9 ± 3.8% (*n* = 9; *p* = 0.0042) by adenosine (**Figures 4G–I**). Hence, our results suggest that adenosine modulates DDI between mitral cells and both granule cells and parvalbumin interneurons.

### Adenosine Inhibits Calcium Currents in Mitral Cells

**FIGURE 5:**
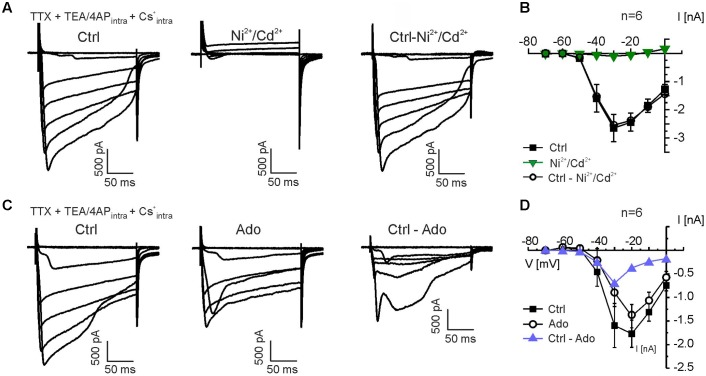
Adenosine inhibits calcium currents in mitral cells. **(A)** Calcium currents were isolated by suppressing sodium currents with TTX (0.5 μM) and potassium currents by replacing K^+^ with Cs^+^ and including TEA (20 mM) and 4-AP (5 mM) in the pipette solution. Calcium currents were entirely blocked by Ni^2+^ (200 μM) and Cd^2+^ (200 μM). Subtraction of currents in presence of Ni^2+^/Cd^2+^ from control currents revealed the Ni^2+^/Cd^2+^-sensitive currents (Ctrl-Ni^2+^/Cd^2+^, right traces). **(B)** Current-voltage (IV) relationship of calcium currents in the absence (Ctrl) and presence of Ni^2+^ and Cd^2+^ (green graph). Ctrl - Ni^2+^/Cd^2+^ represents the current that was blocked by Ni^2+^/Cd^2+^. **(C)** Calcium currents were reduced by adenosine (100 μM). Ctrl - Ado represents the current that was blocked by adenosine. **(D)** IV relationship of the adenosine-sensitive calcium current (blue graph).

To test the inhibition of voltage-gated calcium channels by adenosine, we recorded voltage-sensitive calcium currents in mitral cells by applying voltage steps from -70 to 0 mV. Since K^+^ in the recording pipette solution was replaced by Cs^+^, the pipette solution contained TEA/4-AP, and the recordings were performed in the presence of TTX, isolated calcium currents could be measured. The late phase of some of the calcium current traces appeared to be distorted, presumably due to the high series resistance (9–18 MOhm) and resulting space-clamp problems, whereas the peak current appeared to be less affected by impaired space clamp; hence, the peak current was chosen for analysis. Calcium currents activated around -50 mV, peaked between -30 and -20 mV and were entirely abolished in the presence of Ni^2+^ and Cd^2+^ (*n* = 6) (**Figures 5A,B**). We also tested the effect of adenosine on calcium currents. Application of adenosine reduced calcium currents (**Figure 5C**). The adenosine-sensitive component of the calcium current activated at -40 mV and peaked at -30 mV (**Figure 5D**), suggesting that adenosine receptor activation leads to inhibition of high voltage-activated calcium channels (L-, N- and P/Q-type calcium channels).

**FIGURE 6:**
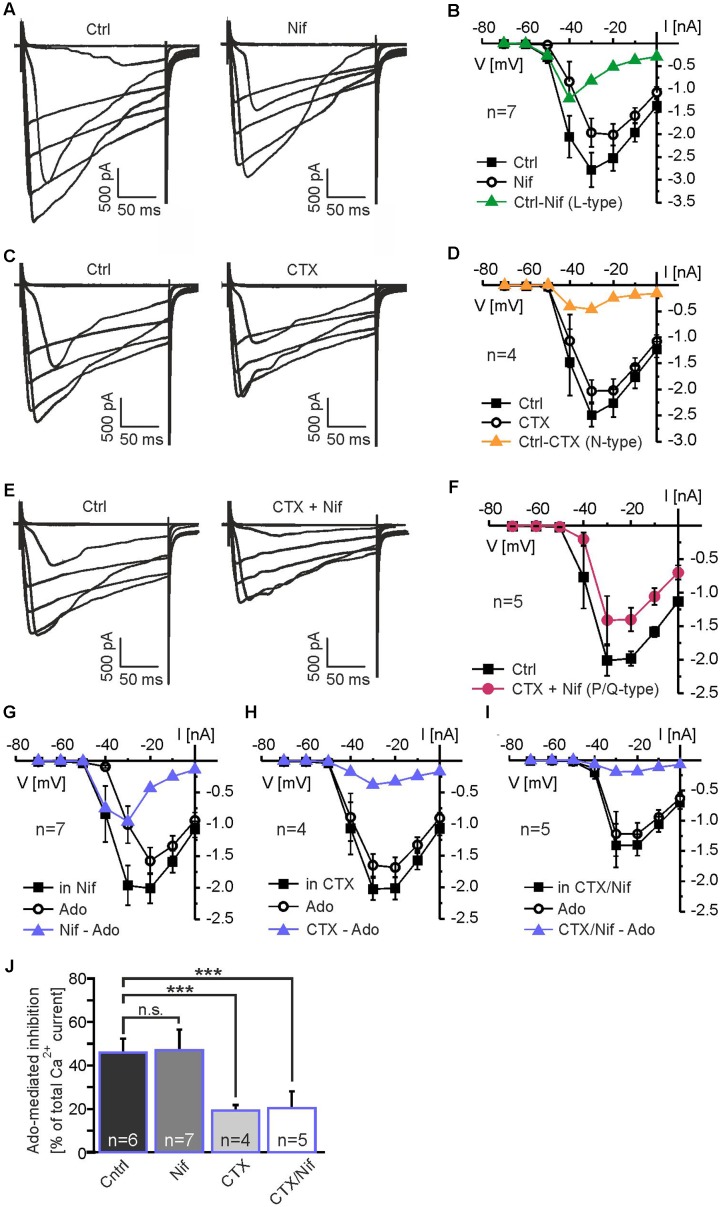
Adenosine inhibits N-type and P/Q-type calcium currents. **(A)** Effect of the L-type calcium channel blocker nifedipine (Nif, 10 μM) on calcium currents. **(B)** IV relationship of nifedipine-sensitive calcium currents (green graph). **(C)** Effect of the N-type calcium channel blocker conotoxin GVIA (CTX, 100 nM) on calcium currents. **(D)** IV relationship of CTX-sensitive calcium currents (yellow graph). **(E)** P/Q-type calcium currents were isolated by blocking N-type and L-type calcium currents with CTX + Nif. **(F)** IV relationship of isolated P/Q-type calcium currents (red graph). **(G)** Adenosine strongly reduces calcium currents in the presence of Nif. **(H)** In the presence of CTX and **(I)** CTX plus Nif, adenosine only weakly reduces calcium currents. **(J)** Effect of adenosine on calcium currents in the absence of calcium channel blockers (Ctrl) and in the presence of Nif, CTX and CTX plus Nif. Incubation with CTX as well as CTX plus Nif significantly reduced the adenosine-mediated attenuation on calcium currents, while Nif alone had no effect on the adenosine-mediated attenuation. n.s., not significant. ^∗∗∗^*p* < 0.001.

To further analyze the contribution of different members of the calcium channel family to calcium currents and to the adenosine-mediated inhibition of calcium currents in mitral cells, we used subtype-specific calcium channel blockers. L-type calcium channels were inhibited by 10 μM nifedipine, which caused a reduction in calcium current amplitude (*n* = 7) (**Figure 6A**). Nifedipine-sensitive calcium currents activated at -50 mV and peaked at -40 mV (**Figure 6B**). The N-type calcium channel blocker conotoxine GVIA (CTX; 100 nM) also reduced the calcium current amplitude (*n* = 4) (**Figure 6C**). The CTX-sensitive calcium currents activated at -40 mV and peaked at -40 to -30 mV (**Figure 6D**). To isolate P/Q-type calcium currents, we blocked L- and N-type calcium currents by co-application of nifedipine and CTX (*n* = 5) (**Figure 6E**). The residual (P/Q-type) calcium current activated at -40 mV and peaked at -30 to -20 mV (**Figure 6F**). We calculated the relative contribution of the different subtypes of voltage-activated calcium currents to the total amount of calcium current of the mitral cell by quantification of the blocker-sensitive currents at a membrane potential of -20 mV, at which the calcium current was largest. We found a contribution of L-type calcium currents to the total calcium current of approximately 21% (*n* = 7), whereas N- and P/Q-type calcium currents contributed 9% (*n* = 4) and 70% (*n* = 5), respectively. It should be noted that due to the space-clamp problems discussed above these values reflect estimations rather than definite values.

In order to determine the calcium channel subtype which is the predominant target of the adenosine-induced inhibition, we measured the effect of adenosine in the absence and in the presence of the calcium channel blockers specified above (**Figures 6G–J**). We analyzed calcium currents elicited by the depolarizing step to -30 mV, at which the adenosine-mediated effect was maximal. In the absence of calcium channel blockers, adenosine reduced the calcium current by 46.1 ± 6.4% (*n* = 6; *p* = 0.049) (**Figures 5D**, **6J**). In presence of L-type calcium channel blocker nifedipine, adenosine reduced the calcium current by 47.1 ± 9.6% (*n* = 7), which was not significantly different from the adenosine-mediated effect in the absence of nifedipine (*p* = 0.89) (**Figures 6G,J**). Hence, L-type calcium channels appear not to be inhibited by adenosine. Blocking N-type calcium channels with CTX, in contrast, significantly reduced the inhibiting effect of adenosine on calcium currents to 19.3 ± 2.5% (*n* = 4; *p* = 0.031) (**Figures 6H,J**), indicating inhibition of N-type calcium channels by adenosine. In addition, P/Q-type calcium currents isolated by a combination of nifedipine and CTX were reduced by 20.3 ± 7.8% (*n* = 5; *p* = 0.025) by adenosine, demonstrating that they were targeted by adenosine receptors. In summary, the adenosine-mediated inhibition of calcium currents was significantly reduced by CTX and by the combination of CTX and nifedipine, while nifedipine alone had no effect (**Figure 6J**). Thus, N- and P/Q-type calcium currents were inhibited by adenosine A_1_ receptors, whereas L-type currents were not affected by adenosine.

### Improved Odor Detection in A_1_ Receptor Knock-out Mice

**FIGURE 7:**
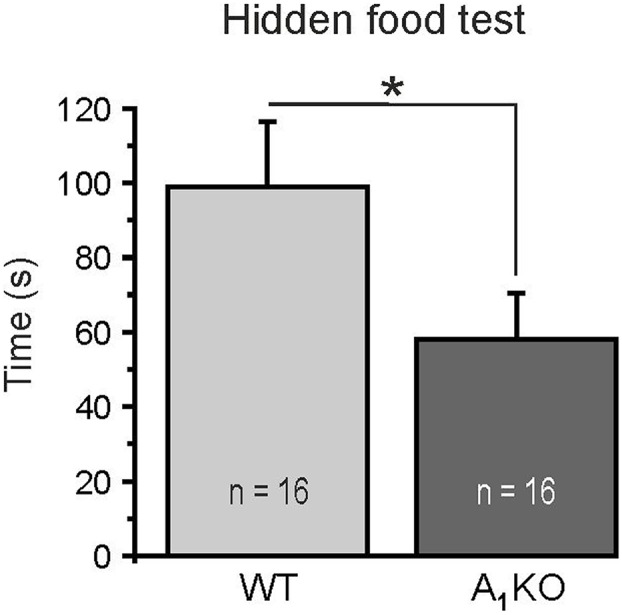
Hidden food test. A_1_ receptor knock-out mice detected a hidden piece of food significantly faster compared to wild-type littermates. ^∗^*p* < 0.05.

We performed a simple odor detection test to assess whether A_1_ receptors affect smelling-related behavior. Animals were placed in a cage with a piece of food hidden in the bedding and the time needed to find the food was measured. Wild-type mice found the food on average after 98.8 ± 17.6 s (*n* = 16) (**Figure 7**). A_1_ receptor knock-out littermates were significantly faster and found the food after 58.0 ± 12.4 s (*n* = 16; *p* = 0.0225). The results suggest that A_1_ receptors might be involved in modulation of odor perception. However, more experiments have to be performed in the future to test whether lack of A_1_ receptor-mediated attenuation of DDI leads to the enhancement of odor detection.

## Discussion

**FIGURE 8:**
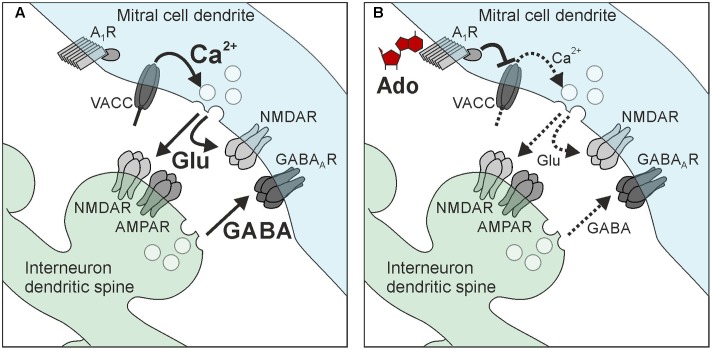
Hypothetical scheme of adenosine-mediated attenuation of reciprocal synapses in mitral cells. **(A)** Recurrent inhibition. **(B)** Adenosine activates A_1_ receptors in mitral cell dendrites, which leads to inhibition of voltage-activated calcium channels (VACC) of the N-type and P/Q-type. The reduction of calcium influx decreases the amount of glutamate released by mitral cells, resulting in reduced excitation of interneurons (granule cells and parvalbumin interneurons) as well as reduced self-excitation. The reduction of interneuron excitation attenuates GABA release and, hence, recurrent inhibition.

In the present study, we analyzed the effects of adenosine on synaptic transmission at reciprocal synapses between mitral cells and interneurons in the olfactory bulb. Activation of adenosine A_1_ receptors attenuated recurrent dendro-dendritic inhibition at synapses between mitral cells and both granule cells and parvalbumin interneurons. This attenuation was accompanied by an inhibition of calcium currents in mitral cells leading to reduced glutamate release from mitral cell dendrites and, hence, less GABA release from interneurons (**Figure 8**). Taken together, this study demonstrates the mechanism of adenosinergic attenuation of neurotransmitter release at reciprocal synapses, which has not been shown before.

### A_1_ Receptors Inhibit Calcium Currents in Mitral Cells

Our results demonstrate a strong inhibition of high voltage-activated calcium currents in mitral cells. The largest fraction of the calcium current was carried by P/Q-type calcium channels (Ca_V_2.1), with moderate contribution of L-type calcium channels (Ca_V_1) and only small contribution of N-type calcium channels (Ca_V_2.2). Despite the low abundance of N-type channels, inhibition of these channels significantly accounted for the adenosine-mediated reduction of calcium currents, suggesting that N-type channels were almost completely inhibited by adenosine. Inhibition of presynaptic N-type currents by A_1_ receptors has also been reported in hippocampal neurons of the CA1 and CA3 regions as well as in retinal ganglion cells, sympathetic neurons and brainstem neurons (Mogul et al., 1993; Zhu and Ikeda, 1993; Umemiya and Berger, 1994; Wu and Saggau, 1994; Sun et al., 2002; Gundlfinger et al., 2007). In hippocampal neurons, inhibition of presynaptic P/Q-type calcium channels also contributed to the A_1_ receptor-mediated attenuation of neurotransmitter release (Gundlfinger et al., 2007). This is in line with our data, since adenosine reduced P/Q-type calcium currents that were isolated by blocking N-type and L-type currents. However, isolated P/Q-type currents were only weakly reduced by adenosine in our experiments. Thus, P/Q-type calcium channels in mitral cells appear to be less sensitive toward adenosinergic inhibition compared to N-type channels, in contrast to hippocampal mossy fiber synapses, in which P/Q-type and N-type calcium channels were inhibited by A_1_ receptors by roughly the same amount (Gundlfinger et al., 2007). Inhibition of L-type calcium channels by A_1_ receptors has been extensively studied in cardiac myocytes and is also present in chemosensory cells of the carotid body (Qu et al., 1993; Belardinelli et al., 1995; Thomas et al., 1998; Rocher et al., 1999). In neurons of the central nervous system, L-type calcium channels appear to be less modulated by A_1_ receptors (Sun et al., 2002; our study). In the brain, L-type channels are predominantly located at somata, dendrites and axons, but not presynaptically (Catterall, 1998; Furukawa, 2013; Zamponi et al., 2015), and, e.g., L-type channels do not mediate synaptic transmission between olfactory receptor neurons and mitral cells (Yuan et al., 2004). Hence, L-type channels appear not suitable for modulation of transmitter release, which may account for the lack of A_1_ receptor-mediated inhibition of L-type channels. Indeed, blocking N-type and P/Q-type calcium channels is sufficient to entirely inhibit dendrodendritic recurrent inhibition in mitral cells, confirming lack of presynaptic L-type channels (Isaacson, 2001). The adenosinergic modulation of synaptic transmission at reciprocal synapses of mitral cells due to N- and P/Q-type calcium channel inhibition appears to be moderate compared with other synapses, at which adenosine inhibits synaptic transmission by far more than 50% (Umemiya and Berger, 1994; Wu and Saggau, 1994; Gundlfinger et al., 2007). Besides high voltage-activated calcium channels, low voltage-activated T-type calcium channels (Ca_V_3) have been demonstrated to contribute to glutamate release from mitral cells (Fekete et al., 2014). However, T-type calcium channels are located in the apical dendrite (Johnston and Delaney, 2010), whereas synapses between mitral cells and granule cells/parvalbumin interneurons that mainly account for the effects studied in the present publication (see Schoppa and Urban, 2003; Uchida et al., 2013) are located at lateral dendrites with low abundance of T-type channels. Hence, the contribution of T-type calcium channels to A_1_ receptor-sensitive calcium currents in mitral cells is considered to be minor.

### A_1_ Receptor Activation Attenuates Recurrent Inhibition

Inhibition of presynaptic calcium influx into mitral cell lateral dendrites by A_1_ receptor activation results in a decrease of glutamate release, which could be measured as a decrease in self-excitation (Aroniadou-Anderjaska et al., 1999; Maher and Westbrook, 2005) and in spontaneous EPSC amplitude in postsynaptic granule cells. Since glutamate release from mitral (and tufted) cells is the main impetus to drive granule cells (Schoppa and Urban, 2003; Egger and Urban, 2006), A_1_ receptor-mediated inhibition of glutamate release from mitral cells leads to a reduction of granule cell activity and hence GABA release, entailing an attenuation of recurrent inhibition of mitral cells (but possibly also lateral inhibition; Geramita et al., 2016). Recurrent inhibition relies on neurotransmitter release on either side of the reciprocal synapse. Our results indicate an inhibitory action of adenosine at the lateral dendrite of mitral cells, however, we cannot exclude that part of the effect takes place at the synaptic counterpart, the granule cell dendritic spine. However, our results render A_1_ receptor-mediated attenuation of GABA release from granule cells and other interneurons unlikely, since GABA release as measured by miniature synaptic currents in mitral cells were not affected by adenosine. Besides mitral cell-to-granule cell synapses, reciprocal synapses between mitral cells and parvalbumin interneurons appear to be subject of adenosinergic attenuation. In contrast to granule cells, which belong to a mono-glomerular column of synaptic connectivity and therefore are supposed to process information in an odor-specific manner (Willhite et al., 2006), parvalbumin interneurons are synaptically connected to mitral cells in a wide, odor non-specific manner and rather act as general modulator of odor information processing (Kato et al., 2013; Miyamichi et al., 2013). Adenosine A_1_ receptors do not discriminate between these two neuronal circuits and thus appear to reduce recurrent inhibition independent of odor-specific synaptic networks. Inhibitory networks in the olfactory bulb are crucial for the establishment of spatio-temporal dynamics and contrast enhancement (Shepherd et al., 2007). Interfering with inhibition in the olfactory bulb has an immediate impact on the performance of the animals in behavioral experiments. Disruption of granule cell-specific GABAergic inhibition in the olfactory bulb network by deletion of the GABA_A_ receptor subunit β3, e.g., leads to a decrease in the ability to discriminate complex odor mixtures (Nusser et al., 2001). By granule cell-specific deletion of the GluA2 subunit, thereby increasing calcium influx in granule cells and, in turn, increasing dendro-dendritic inhibition of mitral cells, the discrimination between related odor mixtures was accelerated in mice (Abraham et al., 2010). A light-induced increase in activity of channelrhodopsin-expressing granule cells improved odor discrimination, while pharmacogenetic inhibition of granule cells impaired odor discrimination (Gschwend et al., 2015). Inhibitory networks not only sharpen odor discrimination, but also have impact on odor detection levels, as shown in experiments in which deletion of a specific subgroup of granule cells (expressing the glycoprotein 5T4) in mice leads to an increased odor detection threshold (Takahashi et al., 2016). Taking the importance of recurrent inhibition in mitral cells into consideration, attenuation of recurrent inhibition by A_1_ receptors potentially has a strong impact on odor information processing. Indeed, in our behavioral experiments, animals that lack A_1_ receptor-mediated attenuation of recurrent inhibition performed better in odor detection, in line with the above mentioned publications that reported improved odor discrimination and lowered detection levels by strong recurrent inhibition of mitral cells as oposed to weak recurrent inhibition (Abraham et al., 2010; Gschwend et al., 2015; Takahashi et al., 2016). It must be noted, however, that the A_1_ receptor knock-out mice we used were conventional knock-out mice and we cannot exclude that the detected effect on smelling behavior is due to lack of A_1_ receptors in another part of the olfactory pathway such as the piriform cortex.

## Conclusion

The relevance of purinergic signaling in the olfactory bulb has gained attention only recently, mainly addressing glial cells. The present study focusses on synaptic transmission and suggests that purinergic neuromodulation might play an important role in adjusting the ratio of excitation and inhibition in the olfactory bulb, thereby affecting odor information processing. The olfactory bulb contains a high number of reciprocal dendro-dendritic synapses and thus serves as a model system to study the physiology of recurrent inhibition such as its purinergic modulation.

## Author Contributions

CL, DH, KS, and NR designed the experiments. DH, JB, KG, KS, and NR performed the experiments and analyzed the data. CL and DH wrote the manuscript. All authors edited the manuscript.

## Conflict of Interest Statement

The authors declare that the research was conducted in the absence of any commercial or financial relationships that could be construed as a potential conflict of interest.

## References

[B1] Abbracchio M. P. Burnstock G. Verkhratsky A. Zimmermann H. (2009). Purinergic signalling in the nervous system: an overview. *Trends Neurosci.* 32 19–29. 10.1016/j.tins.2008.10.001 19008000

[B2] Abraham N. M. Egger V. Shimshek D. R. Renden R. Fukunaga I. Sprengel R. (2010). Synaptic inhibition in the olfactory bulb accelerates odor discrimination in mice. *Neuron* 65 399–411. 10.1016/j.neuron.2010.01.009 20159452 PMC6366558

[B3] Aroniadou-Anderjaska V. Ennis M. Shipley M. T. (1999). Dendrodendritic recurrent excitation in mitral cells of the rat olfactory bulb. *J. Neurophysiol.* 82 489–494. 10.1152/jn.1999.82.1.489 10400976

[B4] Bartel D. L. Rela L. Hsieh L. Greer C. A. (2015). Dendrodendritic synapses in the mouse olfactory bulb external plexiform layer. *J. Comp. Neurol.* 523 1145–1161. 10.1002/cne.23714 25420934 PMC4390432

[B5] Belardinelli L. Shryock J. C. Song Y. Wang D. Srinivas M. (1995). Ionic basis of the electrophysiological actions of adenosine on cardiomyocytes. *FASEB J.* 9 359–365. 7896004 10.1096/fasebj.9.5.7896004

[B6] Bhalla U. S. (2017). Synaptic input sequence discrimination on behavioral time-scales mediated by reaction-diffusion chemistry in dendrites. *Elife* 6:e25827. 10.7554/eLife.25827 28422010 PMC5426902

[B7] Burnstock G. (2013). Introduction to purinergic signalling in the brain. *Adv. Exp. Med. Biol.* 986 1–12. 10.1007/978-94-007-4719-7_1 22879061

[B8] Burnstock G. Fredholm B. B. Verkhratsky A. (2011). Adenosine and ATP receptors in the brain. *Curr. Top. Med. Chem.* 11 973–1011. 10.2174/156802611795347627 21401499

[B9] Catterall W. A. (1998). Structure and function of neuronal Ca^2+^ channels and their role in neurotransmitter release. *Cell Calcium* 24 307–323. 10.1016/S0143-4160(98)90055-0 10091001

[B10] Chen W. R. Shepherd G. M. (2005). The olfactory glomerulus: a cortical module with specific functions. *J. Neurocytol.* 34 353–360. 10.1007/s11068-005-8362-0 16841172

[B11] Chen W. R. Xiong W. Shepherd G. M. (2000). Analysis of relations between NMDA receptors and GABA release at olfactory bulb reciprocal synapses. *Neuron* 25 625–633. 10.1016/S0896-6273(00)81065-X 10774730

[B12] Cheramy A. Leviel V. Glowinski J. (1981). Dendritic release of dopamine in the substantia nigra. *Nature* 289 537–542. 10.1038/289537a0 6258083

[B13] Crespo C. Liberia T. Blasco-Ibanez J. M. Nacher J. Varea E. (2013). The circuits of the olfactory bulb. The exception as a rule. *Anat. Rec.* 296 1401–1412. 10.1002/ar.22732 23907743

[B14] Cunha R. A. (2008). Different cellular sources and different roles of adenosine: A_1_ receptor-mediated inhibition through astrocytic-driven volume transmission and synapse-restricted A_2A_ receptor-mediated facilitation of plasticity. *Neurochem. Int.* 52 65–72. 10.1016/j.neuint.2007.06.026 17664029

[B15] Dias R. B. Rombo D. M. Ribeiro J. A. Henley J. M. Sebastiao A. M. (2013). Adenosine: setting the stage for plasticity. *Trends Neurosci.* 36 248–257. 10.1016/j.tins.2012.12.003 23332692

[B16] Dittman J. S. Regehr W. G. (1996). Contributions of calcium-dependent and calcium-independent mechanisms to presynaptic inhibition at a cerebellar synapse. *J. Neurosci.* 16 1623–1633. 8774431 10.1523/JNEUROSCI.16-05-01623.1996 PMC6578681

[B17] Doengi M. Deitmer J. W. Lohr C. (2008). New evidence for purinergic signaling in the olfactory bulb: A_2A_ and P2Y_1_ receptors mediate intracellular calcium release in astrocytes. *FASEB J.* 22 2368–2378. 10.1096/fj.07-101782 18310463

[B18] Dowling J. E. (1968). Synaptic organization of the frog retina: an electron microscopic analysis comparing the retinas of frogs and primates. *Proc. R. Soc. Lond. B Biol. Sci.* 170 205–228. 10.1098/rspb.1968.0034 4385244

[B19] Droste D. Seifert G. Seddar L. Jädtke O. Steinhäuser C. Lohr C. (2017). Ca^2+^-permeable AMPA receptors in mouse olfactory bulb astrocytes. *Sci. Rep.* 7:44817. 10.1038/srep44817 28322255 PMC5359673

[B20] Dunwiddie T. V. Diao L. Proctor W. R. (1997). Adenine nucleotides undergo rapid, quantitative conversion to adenosine in the extracellular space in rat hippocampus. *J. Neurosci.* 17 7673–7682. 9315889 10.1523/JNEUROSCI.17-20-07673.1997 PMC6793930

[B21] Egger V. Svoboda K. Mainen Z. F. (2003). Mechanisms of lateral inhibition in the olfactory bulb: efficiency and modulation of spike-evoked calcium influx into granule cells. *J. Neurosci.* 23 7551–7558. 12930793 10.1523/JNEUROSCI.23-20-07551.2003 PMC6740749

[B22] Egger V. Urban N. N. (2006). Dynamic connectivity in the mitral cell-granule cell microcircuit. *Semin. Cell Dev. Biol.* 17 424–432. 10.1016/j.semcdb.2006.04.006 16889994

[B23] Fekete A. Johnston J. Delaney K. R. (2014). Presynaptic T-type Ca^2+^ channels modulate dendrodendritic mitral-mitral and mitral-periglomerular connections in mouse olfactory bulb. *J. Neurosci.* 34 14032–14045. 10.1523/JNEUROSCI.0905-14.2014 25319700 PMC6705286

[B24] Fischer T. Rotermund N. Lohr C. Hirnet D. (2012). P2Y1 receptor activation by photolysis of caged ATP enhances neuronal network activity in the developing olfactory bulb. *Purinergic Signal.* 8 191–198. 10.1007/s11302-011-9286-z 22187118 PMC3350580

[B25] Fredholm B. B. IJzerman A. P. Jacobson K. A. Klotz K. N. Linden J. (2001). International Union of Pharmacology. XXV. Nomenclature and classification of adenosine receptors. *Pharmacol. Rev.* 53 527–552. 11734617 PMC9389454

[B26] Friedman D. Strowbridge B. W. (2000). Functional role of NMDA autoreceptors in olfactory mitral cells. *J. Neurophysiol.* 84 39–50. 10.1152/jn.2000.84.1.39 10899181

[B27] Fukunaga I. Herb J. T. Kollo M. Boyden E. S. Schaefer A. T. (2014). Independent control of gamma and theta activity by distinct interneuron networks in the olfactory bulb. *Nat. Neurosci.* 17 1208–1216. 10.1038/nn.3760 24997762 PMC4146518

[B28] Furukawa T. (2013). Types of voltage-gated calcium channels: molecular and electrophysiological views. *Curr. Hypertens. Rev.* 9 170–181. 10.2174/1573402110666140131155912 24479748

[B29] Geramita M. A. Burton S. D. Urban N. N. (2016). Distinct lateral inhibitory circuits drive parallel processing of sensory information in the mammalian olfactory bulb. *Elife* 28:16039. 10.7554/eLife.16039 27351103 PMC4972542

[B30] Gerber U. Greene R. W. Haas H. L. Stevens D. R. (1989). Characterization of inhibition mediated by adenosine in the hippocampus of the rat in vitro. *J. Physiol.* 417 567–578. 10.1113/jphysiol.1989.sp017819 2559967 PMC1189284

[B31] Gschwend O. Abraham N. M. Lagier S. Begnaud F. Rodriguez I. Carleton A. (2015). Neuronal pattern separation in the olfactory bulb improves odor discrimination learning. *Nat. Neurosci.* 18 1474–1482. 10.1038/nn.4089 26301325 PMC4845880

[B32] Gundlfinger A. Bischofberger J. Johenning F. W. Torvinen M. Schmitz D. Breustedt J. (2007). Adenosine modulates transmission at the hippocampal mossy fibre synapse via direct inhibition of presynaptic calcium channels. *J. Physiol.* 582 263–277. 10.1113/jphysiol.2007.132613 17478533 PMC2075290

[B33] Halabisky B. Friedman D. Radojicic M. Strowbridge B. W. (2000). Calcium influx through NMDA receptors directly evokes GABA release in olfactory bulb granule cells. *J. Neurosci.* 20 5124–5134. 10864969 10.1523/JNEUROSCI.20-13-05124.2000 PMC6772283

[B34] Hinds J. W. Hinds P. L. (1976a). Synapse formation in the mouse olfactory bulb. I. Quantitative studies. *J. Comp. Neurol.* 169 15–40. 10.1002/cne.901690103 956463

[B35] Hinds J. W. Hinds P. L. (1976b). Synapse formation in the mouse olfactory bulb. II. Morphogenesis. *J. Comp. Neurol.* 169 41–61. 10.1002/cne.901690104 956464

[B36] Illes P. Nieber K. Norenberg W. (1996). Electrophysiological effects of ATP on brain neurones. *J. Auton. Pharmacol.* 16 407–411. 10.1111/j.1474-8673.1996.tb00064.x 9131427

[B37] Isaacson J. S. (1999). Glutamate spillover mediates excitatory transmission in the rat olfactory bulb. *Neuron* 23 377–384. 10.1016/S0896-6273(00)80787-4 10399942

[B38] Isaacson J. S. (2001). Mechanisms governing dendritic gamma-aminobutyric acid (GABA) release in the rat olfactory bulb. *Proc. Natl. Acad. Sci. U.S.A.* 98 337–342. 10.1073/pnas.021445798 11120892 PMC14591

[B39] Isaacson J. S. Strowbridge B. W. (1998). Olfactory reciprocal synapses: dendritic signaling in the CNS. *Neuron* 20 749–761. 10.1016/S0896-6273(00)81013-2 9581766

[B40] Jackowski A. Parnavelas J. G. Lieberman A. R. (1978). The reciprocal synapse in the external plexiform layer of the mammalian olfactory bulb. *Brain Res.* 159 17–28. 10.1016/0006-8993(78)90106-3 728793

[B41] Jahr C. E. Nicoll R. A. (1982). An intracellular analysis of dendrodendritic inhibition in the turtle *in vitro* olfactory bulb. *J. Physiol.* 326 213–234. 10.1113/jphysiol.1982.sp014187 7108788 PMC1251469

[B42] Johansson B. Halldner L. Dunwiddie T. V. Masino S. A. Poelchen W. Gimenez-Llort L. (2001). Hyperalgesia, anxiety, and decreased hypoxic neuroprotection in mice lacking the adenosine A_1_ receptor. *Proc. Natl. Acad. Sci. U.S.A.* 98 9407–9412. 10.1073/pnas.161292398 11470917 PMC55434

[B43] Johnston J. Delaney K. R. (2010). Synaptic activation of T-type Ca^2+^ channels via mGluR activation in the primary dendrite of mitral cells. *J. Neurophysiol.* 103 2557–2569. 10.1152/jn.00796.2009 20071628

[B44] Kaelin-Lang A. Lauterburg T. Burgunder J. M. (1999). Expression of adenosine A2a receptors gene in the olfactory bulb and spinal cord of rat and mouse. *Neurosci. Lett.* 261 189–191. 10.1016/S0304-3940(99)00022-1 10081981

[B45] Kato H. K. Gillet S. N. Peters A. J. Isaacson J. S. Komiyama T. (2013). Parvalbumin-expressing interneurons linearly control olfactory bulb output. *Neuron* 80 1218–1231. 10.1016/j.neuron.2013.08.036 24239124 PMC3884945

[B46] Ke M. Fujimoto S. Imai T. (2013). SeeDB: a simple and morphology-preserving optical clearing agent for neuronal circuit reconstruction. *Nat. Neurosci.* 16 1154–1161. 10.1038/nn.3447 23792946

[B47] Köles L. Kato E. Hanuska A. Zadori Z. S. Al-Khrasani M. Zelles T. (2016). Modulation of excitatory neurotransmission by neuronal/glial signalling molecules: interplay between purinergic and glutamatergic systems. *Purinergic Signal.* 12 1–24. 10.1007/s11302-015-9480-5 26542977 PMC4749532

[B48] Langer D. Hammer K. Koszalka P. Schrader J. Robson S. Zimmermann H. (2008). Distribution of ectonucleotidases in the rodent brain revisited. *Cell Tissue Res.* 334 199–217. 10.1007/s00441-008-0681-x 18843508

[B49] Linster C. Cleland T. A. (2009). Glomerular microcircuits in the olfactory bulb. *Neural Netw.* 22 1169–1173. 10.1016/j.neunet.2009.07.013 19646847 PMC2771633

[B50] Linster C. Cleland T. A. (2016). Neuromodulation of olfactory transformations. *Curr. Opin. Neurobiol.* 40 170–177. 10.1016/j.conb.2016.07.006 27564660

[B51] Lohr C. Grosche A. Reichenbach A. Hirnet D. (2014). Purinergic neuron-glia interactions in sensory systems. *Pflugers Arch.* 466 1859–1872. 10.1007/s00424-014-1510-6 24705940

[B52] Ludwig M. Pittman Q. J. (2003). Talking back: dendritic neurotransmitter release. *Trends Neurosci.* 26 255–261. 10.1016/S0166-2236(03)00072-9 12744842

[B53] Mahan L. C. McVittie L. D. Smyk-Randall E. M. Nakata H. Monsma F. J. Jr. Gerfen C. R. (1991). Cloning and expression of an A_1_ adenosine receptor from rat brain. *Mol. Pharmacol.* 40 1–7. 1857334

[B54] Maher B. J. Westbrook G. L. (2005). SK channel regulation of dendritic excitability and dendrodendritic inhibition in the olfactory bulb. *J. Neurophysiol.* 94 3743–3750. 10.1152/jn.00797.2005 16107526

[B55] Miyamichi K. Shlomai-Fuchs Y. Shu M. Weissbourd B. C. Luo L. Mizrahi A. (2013). Dissecting local circuits: parvalbumin interneurons underlie broad feedback control of olfactory bulb output. *Neuron* 80 1232–1245. 10.1016/j.neuron.2013.08.027 24239125 PMC3932159

[B56] Mogul D. J. Adams M. E. Fox A. P. (1993). Differential activation of adenosine receptors decreases N-type but potentiates P-type Ca^2+^ current in hippocampal CA3 neurons. *Neuron* 10 327–334. 10.1016/0896-6273(93)90322-I 8382501

[B57] Mombaerts P. (2006). Axonal wiring in the mouse olfactory system. *Annu. Rev. Cell Dev. Biol.* 22 713–737. 10.1146/annurev.cellbio.21.012804.093915 17029582

[B58] Mori K. Kishi K. Ojima H. (1983). Distribution of dendrites of mitral, displaced mitral, tufted, and granule cells in the rabbit olfactory bulb. *J. Comp. Neurol.* 219 339–355. 10.1002/cne.902190308 6619342

[B59] Nicoll R. A. Jahr C. E. (1982). Self-excitation of olfactory bulb neurones. *Nature* 296 441–444. 10.1038/296441a0 6278326

[B60] Nowycky M. C. Mori K. Shepherd G. M. (1981). GABAergic mechanisms of dendrodendritic synapses in isolated turtle olfactory bulb. *J. Neurophysiol.* 46 639–648. 10.1152/jn.1981.46.3.639 7299438

[B61] Nusser Z. Kay L. M. Laurent G. Homanics G. E. Mody I. (2001). Disruption of GABAA receptors on GABAergic interneurons leads to increased oscillatory power in the olfactory bulb network. *J. Neurophysiol.* 86 2823–2833. 10.1152/jn.2001.86.6.2823 11731539

[B62] Okada Y. Sakurai T. Mori M. (1992). Excitatory effect of adenosine on neurotransmission is due to increase of transmitter release in the hippocampal slices. *Neurosci. Lett.* 142 233–236. 10.1016/0304-3940(92)90380-P 1360642

[B63] Pankratov Y. Lalo U. Verkhratsky A. North R. A. (2006). Vesicular release of ATP at central synapses. *Pflugers Arch.* 452 589–597. 10.1007/s00424-006-0061-x 16639550

[B64] Qu Y. Campbell D. L. Whorton A. R. Strauss H. C. (1993). Modulation of basal L-type Ca^2+^ current by adenosine in ferret isolated right ventricular myocytes. *J. Physiol.* 471 269–293. 10.1113/jphysiol.1993.sp019901 8120807 PMC1143962

[B65] Rall W. Shepherd G. M. Reese T. S. Brightman M. W. (1966). Dendrodendritic synaptic pathway for inhibition in the olfactory bulb. *Exp. Neurol.* 14 44–56. 10.1016/0014-4886(66)90023-9 5900523

[B66] Ribeiro J. A. Sebastiao A. M. (2010). Modulation and metamodulation of synapses by adenosine. *Acta Physiol.* 199 161–169. 10.1111/j.1748-1716.2010.02115.x 20345418

[B67] Rieger A. Deitmer J. W. Lohr C. (2007). Axon-glia communication evokes calcium signaling in olfactory ensheathing cells of the developing olfactory bulb. *Glia* 55 352–359. 10.1002/glia.20460 17136772

[B68] Rocher A. Gonzalez C. Almaraz L. (1999). Adenosine inhibits L-type Ca^2+^ current and catecholamine release in the rabbit carotid body chemoreceptor cells. *Eur. J. Neurosci.* 11 673–681. 10.1046/j.1460-9568.1999.00470.x 10051768

[B69] Rombo D. M. Newton K. Nissen W. Badurek S. Horn J. M. Minichiello L. (2015). Synaptic mechanisms of adenosine A_2A_ receptor-mediated hyperexcitability in the hippocampus. *Hippocampus* 25 566–580. 10.1002/hipo.22392 25402014

[B70] Roux L. Madar A. Lacroix M. M. Yi C. Benchenane K. Giaume C. (2015). Astroglial connexin 43 hemichannels modulate olfactory bulb slow oscillations. *J. Neurosci.* 35 15339–15352. 10.1523/JNEUROSCI.0861-15.2015 26586821 PMC6605489

[B71] Salin P. A. Lledo P. M. Vincent J. D. Charpak S. (2001). Dendritic glutamate autoreceptors modulate signal processing in rat mitral cells. *J. Neurophysiol.* 85 1275–1282. 10.1152/jn.2001.85.3.1275 11247996

[B72] Sassoe-Pognetto M. (2011). Molecular and functional heterogeneity of neural circuits: an example from the olfactory bulb. *Brain Res. Rev.* 66 35–42. 10.1016/j.brainresrev.2010.06.003 20600309

[B73] Schoppa N. E. Kinzie J. M. Sahara Y. Segerson T. P. Westbrook G. L. (1998). Dendrodendritic inhibition in the olfactory bulb is driven by NMDA receptors. *J. Neurosci.* 18 6790–6802. 9712650 10.1523/JNEUROSCI.18-17-06790.1998 PMC6792983

[B74] Schoppa N. E. Urban N. N. (2003). Dendritic processing within olfactory bulb circuits. *Trends Neurosci.* 26 501–506. 10.1016/S0166-2236(03)00228-5 12948662

[B75] Sebastiao A. M. Cunha R. A. Cascalheira J. F. Ribeiro J. A. (1999). Adenine nucleotides as inhibitors of synaptic transmission: role of localised ectonucleotidases. *Prog. Brain Res.* 120 183–192. 10.1016/S0079-6123(08)63555-4 10550997

[B76] Segal M. (1982). Intracellular analysis of a postsynaptic action of adenosine in the rat hippocampus. *Eur. J. Pharmacol.* 79 193–199. 10.1016/0014-2999(82)90625-2 7094996

[B77] Shepherd G. M. Chen W. R. Willhite D. Migliore M. Greer C. A. (2007). The olfactory granule cell: from classical enigma to central role in olfactory processing. *Brain Res. Rev.* 55 373–382. 10.1016/j.brainresrev.2007.03.005 17434592

[B78] Sun X. Barnes S. Baldridge W. H. (2002). Adenosine inhibits calcium channel currents via A_1_ receptors on salamander retinal ganglion cells in a mini-slice preparation. *J. Neurochem.* 81 550–556. 10.1046/j.1471-4159.2002.00832.x 12065663

[B79] Takahashi H. Ogawa Y. Yoshihara S. Asahina R. Kinoshita M. Kitano T. (2016). A subtype of olfactory bulb interneurons is required for odor detection and discrimination behaviors. *J. Neurosci.* 36 8210–8227. 10.1523/JNEUROSCI.2783-15.2016 27488640 PMC6601955

[B80] Thomas G. P. Sims S. M. Cook M. A. Karmazyn M. (1998). Hydrogen peroxide-induced stimulation of L-type calcium current in guinea pig ventricular myocytes and its inhibition by adenosine A_1_ receptor activation. *J. Pharmacol. Exp. Ther.* 286 1208–1214. 9732380

[B81] Thyssen A. Hirnet D. Wolburg H. Schmalzing G. Deitmer J. W. Lohr C. (2010). Ectopic vesicular neurotransmitter release along sensory axons mediates neurovascular coupling via glial calcium signaling. *Proc. Natl. Acad. Sci. U.S.A.* 107 15258–15263. 10.1073/pnas.1003501107 20696909 PMC2930556

[B82] Thyssen A. Stavermann M. Buddrus K. Doengi M. Ekberg J. A. St John J. A. (2013). Spatial and developmental heterogeneity of calcium signaling in olfactory ensheathing cells. *Glia* 61 327–337. 10.1002/glia.22434 23109369

[B83] Toida K. (2008). Synaptic organization of the olfactory bulb based on chemical coding of neurons. *Anat. Sci. Int.* 83 207–217. 10.1111/j.1447-073X.2008.00247.x 19159348

[B84] Trussell L. O. Jackson M. B. (1985). Adenosine-activated potassium conductance in cultured striatal neurons. *Proc. Natl. Acad. Sci. U.S.A.* 82 4857–4861. 10.1073/pnas.82.14.4857 2991897 PMC391004

[B85] Uchida N. Eshel N. Watabe-Uchida M. (2013). Division of labor for division: inhibitory interneurons with different spatial landscapes in the olfactory system. *Neuron* 80 1106–1109. 10.1016/j.neuron.2013.11.013 24314722 PMC4175561

[B86] Umemiya M. Berger A. J. (1994). Activation of adenosine A_1_ and A_2_ receptors differentially modulates calcium channels and glycinergic synaptic transmission in rat brainstem. *Neuron* 13 1439–1446. 10.1016/0896-6273(94)90429-4 7993635

[B87] Urban N. N. Castro J. B. (2010). Functional polarity in neurons: What can we learn from studying an exception? *Curr. Opin. Neurobiol.* 20 538–542. 10.1016/j.conb.2010.07.005 20724138 PMC2946436

[B88] Willhite D. C. Nguyen K. T. Masurkar A. V. Greer C. A. Shepherd G. M. Chen W. R. (2006). Viral tracing identifies distributed columnar organization in the olfactory bulb. *Proc. Natl. Acad. Sci. U.S.A.* 103 12592–12597. 10.1073/pnas.0602032103 16895993 PMC1567923

[B89] Wirkner K. Gerevich Z. Krause T. Gunther A. Koles L. Schneider D. (2004). Adenosine A_2A_ receptor-induced inhibition of NMDA and GABA_A_ receptor-mediated synaptic currents in a subpopulation of rat striatal neurons. *Neuropharmacology* 46 994–1007. 10.1016/j.neuropharm.2004.01.008 15081796

[B90] Wu L. G. Saggau P. (1994). Adenosine inhibits evoked synaptic transmission primarily by reducing presynaptic calcium influx in area CA1 of hippocampus. *Neuron* 12 1139–1148. 10.1016/0896-6273(94)90321-2 8185949

[B91] Yang M. Crawley J. N. (2009). Simple behavioral assessment of mouse olfaction. *Curr. Protoc. Neurosci.* 48 8.24.1–8.24.12. 10.1002/0471142301.ns0824s48 19575474 PMC2753229

[B92] Yawo H. Chuhma N. (1993). Preferential inhibition of omega-conotoxin-sensitive presynaptic Ca^2+^ channels by adenosine autoreceptors. *Nature* 365 256–258. 10.1038/365256a0 8396730

[B93] Yokoi M. Mori K. Nakanishi S. (1995). Refinement of odor molecule tuning by dendrodendritic synaptic inhibition in the olfactory bulb. *Proc. Natl. Acad. Sci. U.S.A.* 92 3371–3375. 10.1073/pnas.92.8.3371 7724568 PMC42168

[B94] Yuan Q. Mutoh H. Debarbieux F. Knöpfel T. (2004). Calcium signaling in mitral cell dendrites of olfactory bulbs of neonatal rats and mice during olfactory nerve Stimulation and beta-adrenoceptor activation. *Learn. Mem.* 11 406–411. 10.1101/lm.75204 15286182 PMC498321

[B95] Zamponi G. W. Striessnig J. Koschak A. Dolphin A. C. (2015). The physiology, pathology, and pharmacology of voltage-gated calcium channels and their future therapeutic potential. *Pharmacol. Rev.* 67 821–870. 10.1124/pr.114.009654 26362469 PMC4630564

[B96] Zhang W. Linden D. J. (2009). Neuromodulation at single presynaptic boutons of cerebellar parallel fibers is determined by bouton size and basal action potential-evoked Ca transient amplitude. *J. Neurosci.* 29 15586–15594. 10.1523/JNEUROSCI.3793-09.2009 20007482 PMC2829188

[B97] Zhang X. Chen Y. Wang C. Huang L. M. (2007). Neuronal somatic ATP release triggers neuron-satellite glial cell communication in dorsal root ganglia. *Proc. Natl. Acad. Sci. U.S.A.* 104 9864–9869. 10.1073/pnas.0611048104 17525149 PMC1887586

[B98] Zhu Y. Ikeda S. R. (1993). Adenosine modulates voltage-gated Ca^2+^ channels in adult rat sympathetic neurons. *J. Neurophysiol.* 70 610–620. 10.1152/jn.1993.70.2.610 8410161

[B99] Zimmermann H. (2000). Extracellular metabolism of ATP and other nucleotides. *Naunyn Schmiedebergs Arch. Pharmacol.* 362 299–309. 10.1007/s002100000309 11111825

[B100] Zimmermann H. Zebisch M. Sträter N. (2012). Cellular function and molecular structure of ecto-nucleotidases. *Purinergic Signal.* 8 437–502. 10.1007/s11302-012-9309-4 22555564 PMC3360096

