# Supplementary material for: Adenosine A1 Receptor-Mediated Attenuation of Reciprocal Dendro-Dendritic Inhibition in the Mouse Olfactory Bulb
Source: Front Cell Neurosci. 2018 Jan 15;11:435. doi: 10.3389/fncel.2017.00435 (PMC5775233; doi:10.3389/fncel.2017.00435)
Supplement: Supplementary file 1 [file Data_Sheet_1.docx]

Supplementary Material

Adenosine A_1_ receptor-mediated attenuation of reciprocal dendro-dendritic inhibition in the mouse olfactory bulb

**Kristina Schulz, Natalie Rotermund, Katarzyna Grzelka, Jan Benz, Christian Lohr*, Daniela Hirnet***

*** Correspondence:** Christian Lohr: christian.lohr@uni-hamburg.de and Daniela Hirnet: daniela.hirnet@uni-hamburg.de


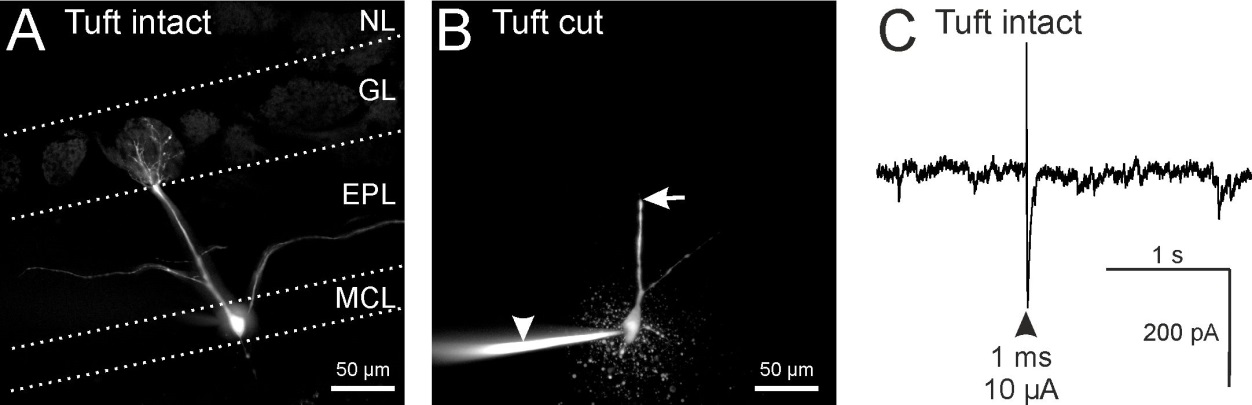


**Supplementary Figure 1.** Alexa Fluor594-filled mitral cells with (**A**) intact apical tuft and (**B**) apical tuft that was cut (arrow) during the procedure of brain slice preparation. The arrowhead points to the Alexa 594-filled patch-clamp pipette. EPL, external plexiform layer; GL, glomerular layer; MCL, mitral cell layer; NL, nerve layer. (**C**) Single-pulse stimulation (arrowhead) of sensory axons in the nerve layer evoked an EPSC in an intact mitral cell, demonstrating successful recording of synaptic currents from the apical tuft.


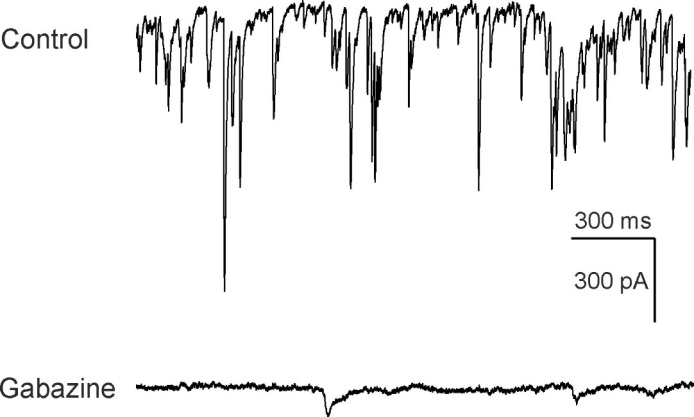


**Supplementary Figure 2.** Spontaneous synaptic events in mitral cells. Numerous synaptic currents were recorded in a mitral cell in the absence of synaptic blockers (upper trace). In the presence of the GABA_A_ receptor blocker gabazine (10 µM), the frequency of synaptic events was greatly reduced (lower trace), indicating that the majority of spontaneous synaptic events were GABAergic. GABAergic inhibitory synaptic events were inwardly directed due to the high chloride concentration in the pipette solution.
